# Personalized prescription of imatinib in recurrent granulosa cell tumor of the ovary: case report

**DOI:** 10.1101/mcs.a003434

**Published:** 2019-04

**Authors:** Elena V. Poddubskaya, Madina P. Baranova, Daria O. Allina, Marina I. Sekacheva, Lyudmila A. Makovskaia, Dmitriy E. Kamashev, Maria V. Suntsova, Viktoria S. Barbara, Irina N. Kochergina-Nikitskaya, Alexey A. Aleshin

**Affiliations:** 1 Clinical Center Vitamed, Moscow, 121309, Russia;; 2 I.M. Sechenov First Moscow State Medical University (Sechenov University), Moscow, 119991, Russia;; 3 FSBEI FPE Russian Medical Academy of Continuing Professional Education MOH, Moscow, 125993, Russia;; 4 Department of Pathology, Morozov Children's City Hospital, Moscow, 119049, Russia;; 5 Faculty of Fundamental Medicine, Lomonosov Moscow State University, Moscow, 119991, Russia;; 6 Shemyakin–Ovchinnikov Institute of Bioorganic Chemistry, Moscow, 117997, Russia;; 7 Oncological Dispensary of the Republic of Karelia, Petrozavodsk, 185002, Russia;; 8 The Institute of General Pathology and Pathophysiology, Moscow, 125315, Russia;; 9 Stanford University School of Medicine, Stanford, California 94305, USA

**Keywords:** ovarian neoplasm

## Abstract

Ovarian cancer is the fifth leading cause of cancer-related female mortality and the most lethal gynecological cancer. In this report, we present a rare case of recurrent granulosa cell tumor (GCT) of the ovary. We describe the case of a 26-yr-old woman with progressive GCT of the right ovary despite multiple lines of therapy who underwent salvage therapy selection based on a novel bioinformatical decision support tool (Oncobox). This analysis generated a list of potentially actionable compounds, which when used clinically lead to partial response and later long-term stabilization of the patient's disease.

## INTRODUCTION

Ovarian cancer is the fifth most common cause of cancer-related death among women and the most lethal gynecological malignancy (Stewart and Wild 2014). Worldwide, malignant ovarian neoplasms account for an estimated 225,500 new cases and 140,200 deaths (22,300 and 15,500, respectively in the United States) (Siegel et al. 2017). Total incidence has increased by 6% from 2005 to 2010 (Siegel et al. 2017). Despite significant advances in the development of new treatment regimens, the survival rate has remained poor with 50% of affected women succumbing to their disease at 5 years (Eisenhauer 2017).

Granulosa cell tumor (GCT) of the ovary constitutes 2%–5% of all ovarian malignancies (Schumer and Cannistra 2003). Most cases are diagnosed early, and the prognosis is favorable (Khosla et al. 2014). The scope of surgical treatment depends on the stage and age of the patient. In cases of favorable prognosis and reproductive age, the treatment may be limited to unilateral salpingo-oophorectomy and further observation. In postmenopausal women, bilateral salpingo-oophorectomy is recommended. The proper amount of surgical intervention in GCT is extirpation of the uterus with appendages and removal of the large omentum. At late (II–IV) stages of the disease, a radical tumor removal is necessary.

Surgical treatment (radical removal of recurrent tumors or cytoreductive operations) is also recommended by NCCN for treatment of GCT relapses and metastases. Common metastatic lesions include neoplasms in both the pelvic area and the parenchymal organs.

Adjuvant platinum-based chemotherapy is recommended for patients with a high risk of recurrence. In the presence of residual neoplasms, regimens that include platinum drugs are effective in a considerable proportion of cases (∼60%) (Bridgewater and Rustin 1999). In this report, we present a case of recurrent ovarian GCT, which progressed during platinum-based therapy but was successfully treated with imatinib monotherapy. The imatinib prescription was based on individual analysis of gene expression in the patient's tumor and bioinformatic profiling of signaling pathway activation.

## RESULTS

A 26-yr-old woman was diagnosed at N.N. Blokhin Russian Cancer Research Center with primary GCT of the right ovary in 2001. The patient underwent unilateral salpingo-oophrectomy, with peritoneal biopsies without evidence of tumor growth in the left ovary and solitary complexes of malignant cells. From 2003 until 2008, the patient underwent three excisions of the following neoplasms: cystadenoma in the left ovary (the operation was organo-preserving because of pregnancy planning, and part of the left ovary was saved), cystic formation in the left ovary, and GCT in the right lateral region of the abdominal cavity.

Dissemination of neoplastic foci on the patient's peritoneum was observed in 2010. Ultrasound examination revealed a 2.9 × 0.8-cm neoplasm in the S7 liver capsule; the dimensions of cystic formation in the pelvis were 4.0 × 3.2 × 2.6 cm. The patient received megestrol (Megace, 160 mg/day) for 5 mo; however, lesions progressed during this period. Extirpation of recurrent tumors in the pelvic area was performed with dissection of adhesions and resection of the large omentum.

**Figure 1.**
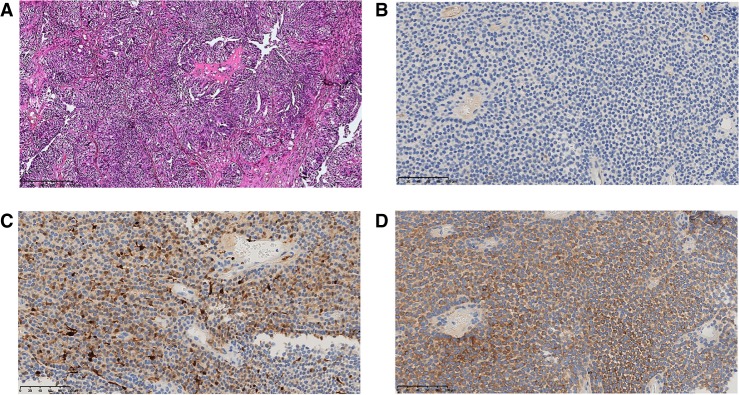
(*A*) Hematoxylin and eosin staining shows granulosa cell ovarian cancer (magnification, 200×); (*B*) c-*Kit* IHC (Cell Marque, YR145) demonstrated the tumor cells to be negative; (*C*) calretinin (marker of ovarian sex cord-stromal tumor) IHC (Roche, Ventana, SP65) demonstrated the tumor cells to be positive; (*D*) inhibin (marker of ovarian sex cord-stromal tumor) IHC (Cell Marque, R1) demonstrated the tumor cells to be positive.

Relapse and further progression of the disease started in 2012. Abdominal examinations showed formations in the Douglas space, in the pelvic area and to the left behind the uterus, in the field of the splenic hilum, and in the lateral canal of the liver's right lobe. We introduce enumeration of neoplasms for further comparison of the measurements. The dimensions of all neoplasms across the study are summarized in Supplemental Table S1. The largest formations were identified on the posterior surface of the liver (3.8 × 2.5 cm, neoplasm #1) and in the right part of the posterior paranephric fat (6.8 × 5.8 cm, neoplasm #3). A 4.8 × 4.5-cm neoplasm in the Douglas space (neoplasm #2) displaced the rectum to the right. The patient underwent cytoreductive (debulking) surgery. Hematoxylin and eosin staining confirmed the primary origin of the tumor (Fig. 1). The patient’s condition after the operation was found satisfactory without complications. BEP (bleomycin, etoposide, and cisplatin) therapy was prescribed following the surgical procedures; however, it was the patient's decision to refuse further chemotherapy.

The disease progressed in 2014. Lesions revealed included multiple cystic neoplasms in the right lobe of the liver, neoplasms in the splenic hilum and in the epigastrium, and multiple lesions in the navel field. The neoplasm in the pelvic area progressed. The patient agreed to receive chemotherapy in the beginning of 2015 and was administered four courses of BEP therapy. However, ultrasound examination revealed continuous progression of the disease.

**Figure 2.**
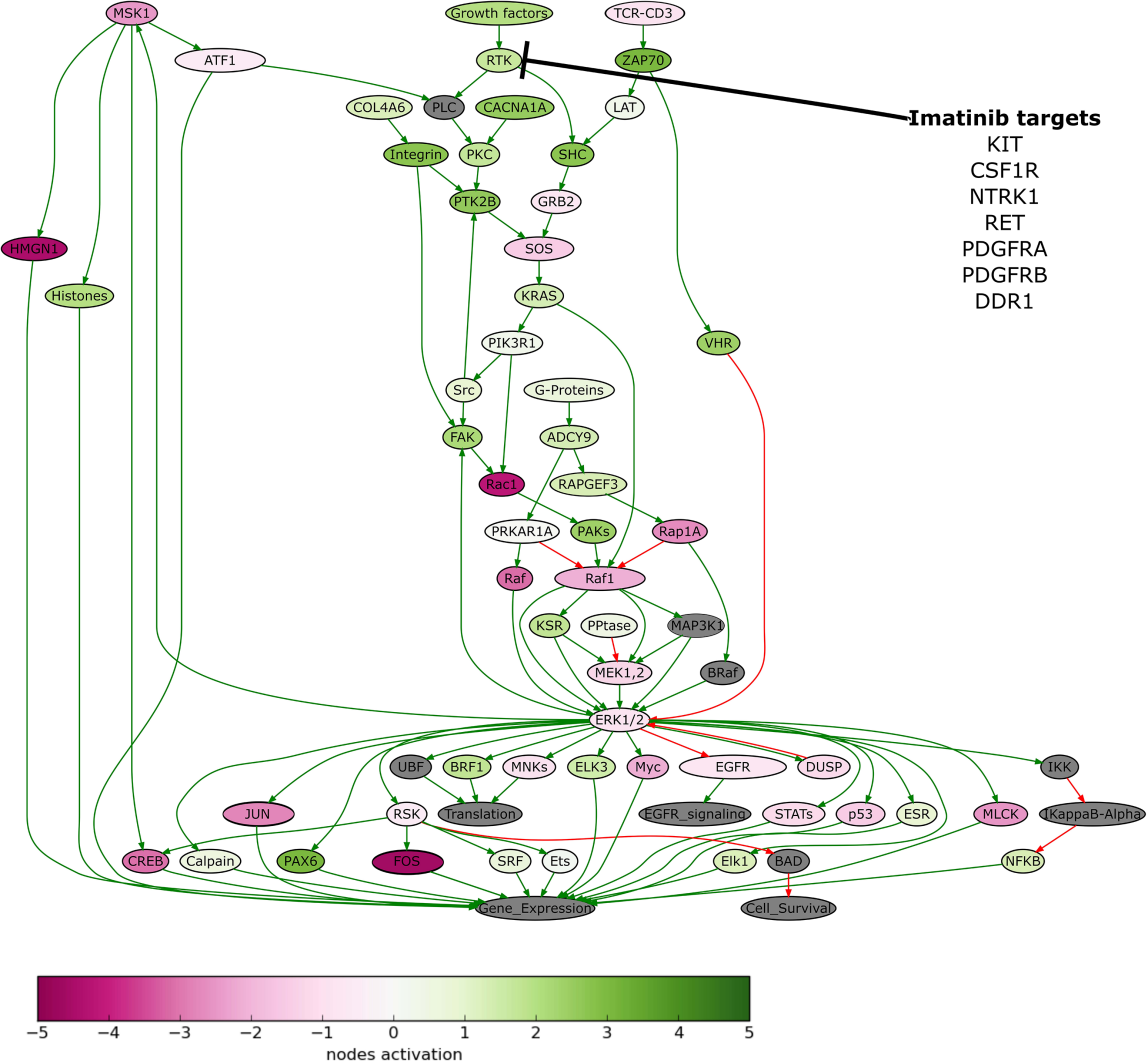
The ERK signaling pathway was hyperactivated in the patient's tumor tissue. Visualization was provided by the Oncobox software. The pathway is shown as an interacting network, where green arrows indicate activation, and red arrows indicate inhibition. The color depth of each node of the network corresponds to the logarithms of the case-to-normal (CNR) expression rate for each node, in which “normal” is a geometric average between normal tissue samples; the scale represents the extent of up-/down-regulation. The molecular targets of imatinib are shown by black arrows.

To identify further treatment options, we performed molecular analysis of the patient's tumor. We extracted DNA from the patients’ formalin-fixed, paraffin-embedded (FFPE) tumor tissue sample, obtained following cytoreductive (debulking) surgery in 2013, and performed whole-exome sequencing. The sequencing data were deposited in the NCBI Sequence Read Archive (SRA) under accession ID PRJNA503667. The tumor appeared to be FOXL2 C134W-positive, which corresponds to the adult-type GCT of the ovary (Shah et al. 2009). We also extracted RNA from the patients’ sample and profiled gene expression (for details, see Materials and Methods section). The results of molecular analysis were deposited to the Gene Expression Omnibus (GEO) database under accession ID GSE112579. We next used the Oncobox bioinformatical platform for personalized prescription of target therapy. The Oncobox target drug scoring algorithm is based on the analysis of the intracellular signaling pathway activation using gene expression data. Oncobox analysis estimates activation level for approximately 380 cancer-related signaling pathways. In particular, Oncobox analysis revealed that ERK signaling was one of the pathways, which was strongly up-regulated in the patient's tumor sample, as compared to the normal tissue taken from unrelated postmortal donors (Fig. 2). According to the results of the Oncobox test, the following target drugs could be potentially effective for treatment of this patient (in a decreasing efficiency order): regorafenib, sorafenib, sunitinib, pazopanib, axitinib, aflibercept, cabozantinib, and imatinib. The pathway activation profiles and the full ratings of the target drugs are provided in Supplemental Table S2. We also determined the expression level of c-*Kit* (Imatinib target) using immunohistochemistry. The sample appeared to be c-*Kit*-negative, in accordance with microarray data (Table 1; Supplemental Table S3). The patient was administered sorafenib (Nexavar, 400 mg daily) from October 2015. The expression level of sorafenib target genes is presented in Table 2. However, sorafenib was not well tolerated and the patient developed polyarthritis. Sorafenib therapy was terminated 2 mo after initial administration. In January 2016, ultrasound examination indicated a decrease in the size of several cystic formations: Three out of four neoplasms decreased in size after sorafenib treatment (7% decrease in the sum of all lesions' diameters).

**Table 1.** Expression levels (log_2_ fold change compared with normal ovarian tissue) for sorafenib-targeted genes

Symbol	Log_2_ (fold change)
*FGFR1*	0.604536
*FLT1*	−0.949541
*FLT3*	0.782428
*FLT4*	2.434109
*KDR*	4.068401
*KIT*	−3.92423
*PDGFRB*	−1.72703
*RAF1*	−3.010054
*RET*	4.569097

**Table 2.** Expression levels (log_2_ fold change compared with normal ovarian tissue) for imatinib-targeted genes

Symbol	Log_2_ (fold change)
*ABL1*	−3.04638
*CSF1R*	2.749138
*KIT*	−3.92423
*NTRK1*	3.338048
*PDGFRA*	−0.97151
*PDGFRB*	−1.72703
*RET*	4.569097

**Figure 3.**
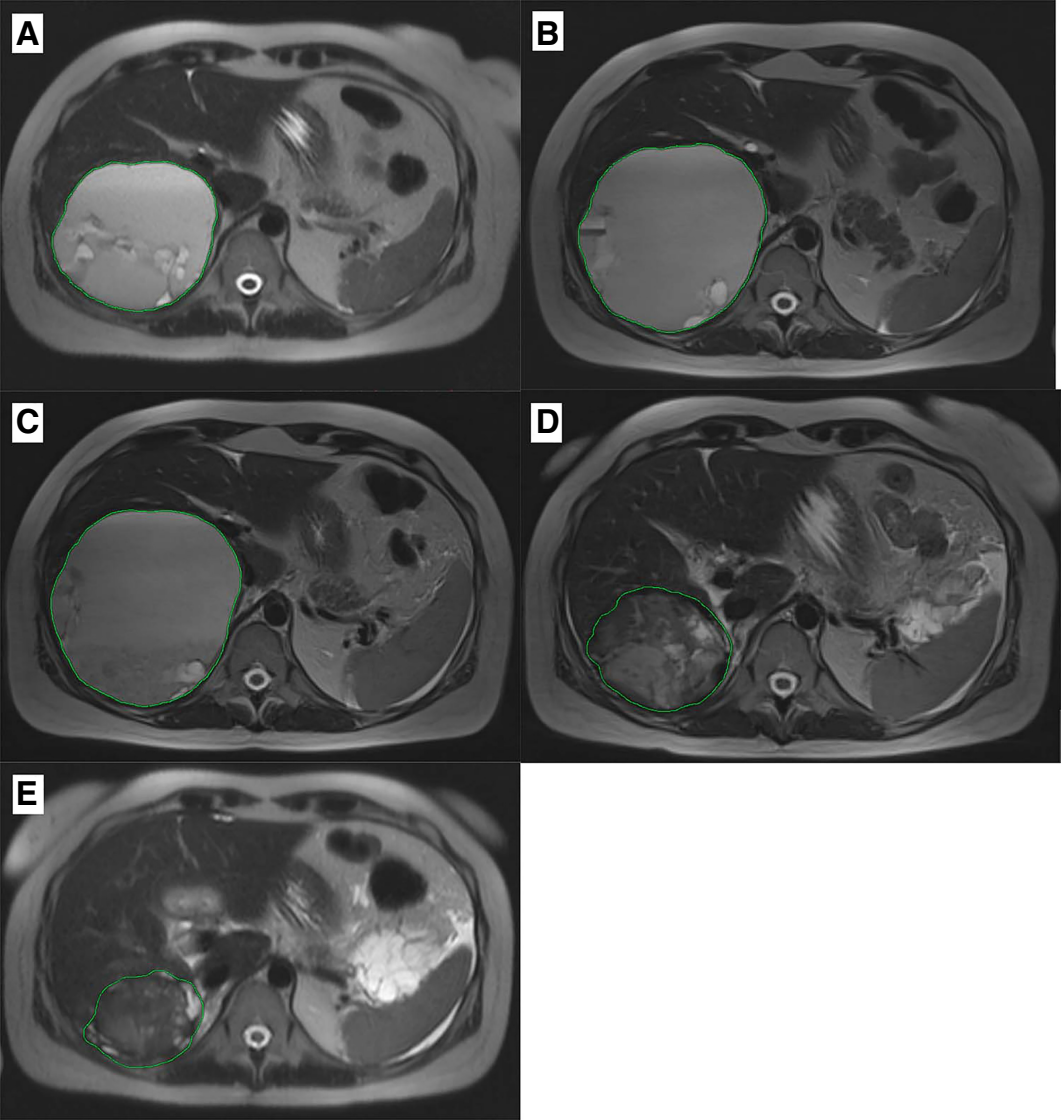
Cystic-solid neoplasm on the lower contour of the liver (neoplasm #1). (*A*) MRI from 03.2016; (*B*) MRI from 06.2016; (*C*) MRI from 09.2016; (*D*) MRI from 10.2017; (*E*) MRI from 03.2018.

**Figure 4.**
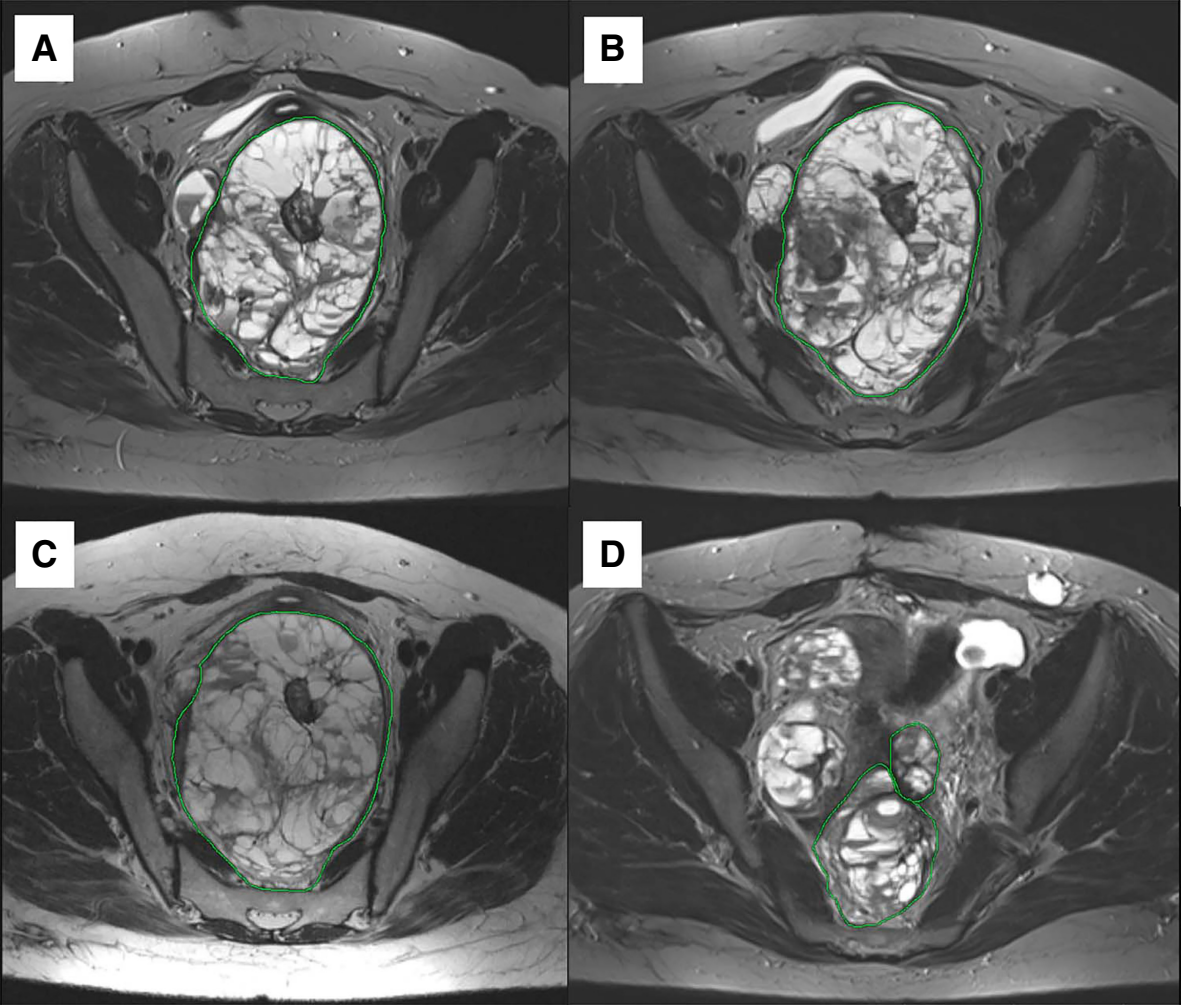
Cystic-solid multinodular formation in Douglas space, part of nodules with high-density content (Neoplasm #2). (*А*) MRI from 03.2016; (*B*) MRI from 06.2016; (*C*) MRI from 09.2016; (*D*) MRI from 03.2018.

**Figure 5.**
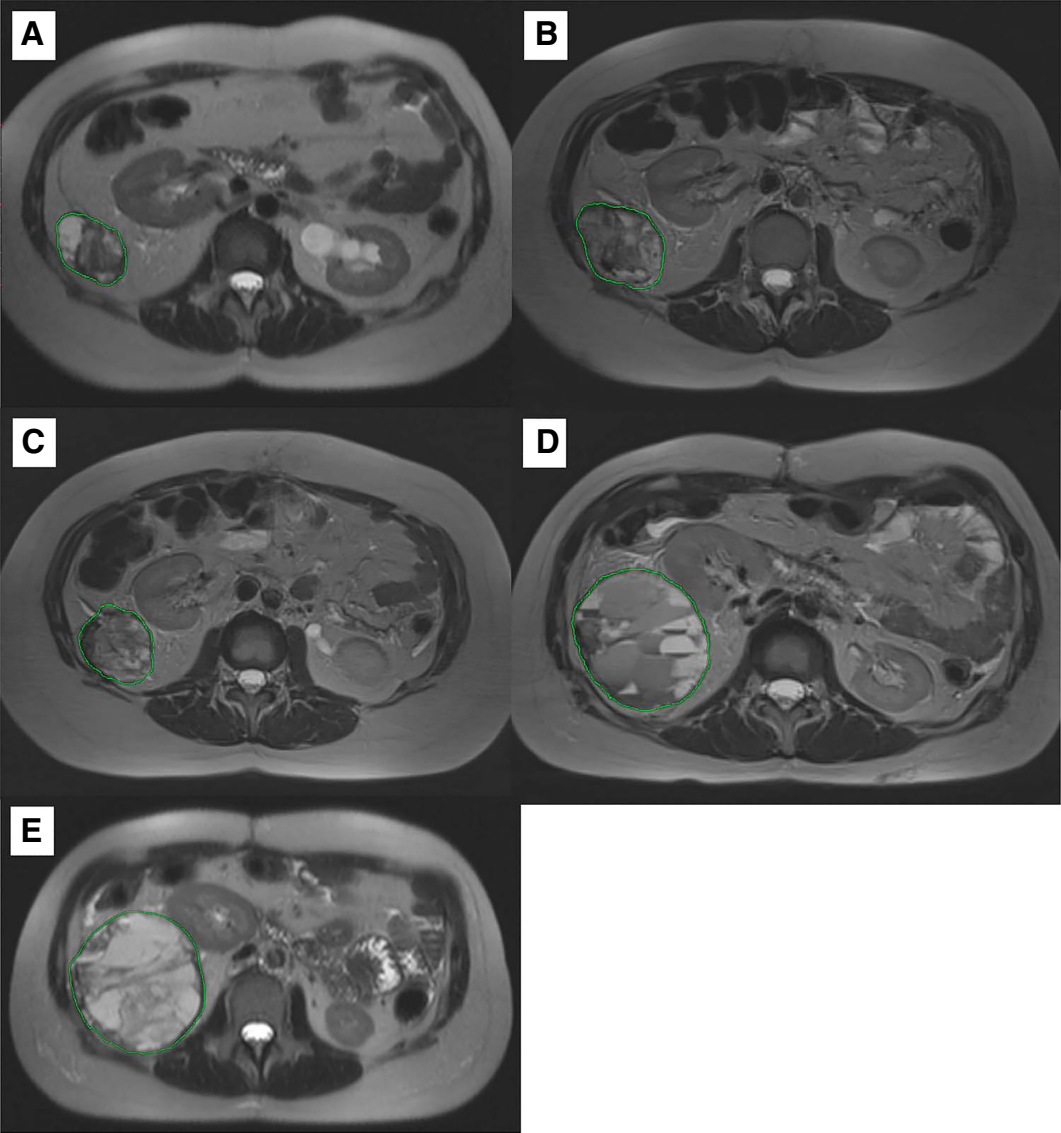
Cystic-solid neoplasm with tuberous contours in the right lateral channel (neoplasm #3). (*А*) MRI from 03.2016; (*B*) RI from 06.201; (*C*) MRI from 09.2016; (*D*) MRI from 10.2017; (*E*) MRI from 03.2018.

As sorafenib was not tolerated by the patient, therapy regimen was switched to imatinib—another TKI, which was predicted to be effective for this patient using the Oncobox test. Imatinib (400 mg daily) was administered from February until May 2016. An MRI examination in March 2016 revealed a slight increase in sizes of the neoplasms. Neoplasm #1, located in the capsule of the right lobe of the liver, was 12.0 × 10.4 × 10.2 cm in size (Fig. 3); in the right lateral channel, 5.3 × 3.3 × 4.5 cm (neoplasm #3; Fig. 5); in the splenic hilum, 3.5 × 1.6 cm in size (neoplasm #4; Supplemental Fig. S1); the formation in the Douglas space in the pelvis decreased in size to 12.7 × 8.7 cm (neoplasm #2; Fig. 4).

From May until July 2016, imatinib was replaced by the filachromine (generic Imatinib) because of the unavailability of original Imatinib (Gleevec). MRI examination in June indicated stabilization of the disease (Supplemental Table S1; Figs. 3–5A,B). However, generic imatinib was not as well tolerated as the original imatinib (Gleevec). The patient complained of severe colitis. Filachromine administration was terminated because of this side effect. Indeed, substitution of the original drug with a generic could potentially decrease the efficacy of the treatment, as it was previously observed with another imatinib generic—Cipla (Mattar 2010). From August until October, the patient did not receive any therapy. MRI performed in September confirmed stabilization of the patient's state (Supplemental Table S1; Figs. 3–5B,C); the sum of all target lesions' diameters increased by 1.6%. However, ascites, compression of the left ureter, and blockage of the left kidney were observed.

MRI of the abdomen and pelvic area performed in December 2016 (source images were not available) revealed that the cystic-solid nodules under the right lobe of the liver (13.5 × 12.0 cm) squeeze the liver and liver’s gates; the nodules in the right lateral canal (7.8 × 5.5 cm) displaced the right kidney upward and medially. The dimensions of the tumor in the pelvic cavity were 17.0 × 11.0 cm, the rectum was shifted to the right and compressed, and the bladder was compressed and shifted anteriorly.

Another cytoreductive surgery was performed in December 2016. Neoplasms in the right lobe of the liver (#1) and in the pelvic area (#5) were partially removed. Revision of the abdominal cavity revealed significant adhesive process. Tumor nodules with a thin capsule were intimately connected with the bladder, rectum, and ureters. Operative blood loss was 4 L. The metastatic nodules were removed in part because of technical difficulties. The ultrasound examination performed after surgery revealed that the liver was enlarged with the right lobe pushed back and pressed by a cystic neoplasm (neoplasm #1, 11.0 × 12.0 × 14.0 cm). Similar neoplasm of size 8.4 × 6.6 cm (neoplasm #3) was located laterally.

Original imatinib (Gleevec) became available and its administration (400 mg daily) started in February 2017. Before imatinib treatment, ultrasound examination in February 2017 revealed a tumor in the region of the posterolateral liver capsule (15.5 × 12.0 cm, neoplasm #1), in the splenic hilum (5.0 × 3.3 cm, neoplasm #4), and in the area of the lateral channel in the abdominal cavity (9.0 × 7.5 cm, neoplasm #3) and several neoplasms in the pelvic region (neoplasms #7, #8, #9). MRI analysis in October 2017 revealed a decrease in size for these lesions, with the exception of the neoplasm in the splenic hilum area (its size was stable) (Supplemental Table S1). A new cystic neoplasm (#12, Supplemental Fig. S9) was observed during MRI examination in October. The sum of the largest diameters for all target lesions increased by 12% when compared to the MRI results from September 2016 (considering only the lesions investigated during both examinations), which supports disease stabilization.

An MRI examination in March 2018 revealed moderate growth of lesions #3, #4, #10, and #13 (Fig. 5; Supplemental Figs. S1, S7, and S10, respectively; sum of largest diameters for all target lesions increased by 15%), which corresponds to disease stabilization. As of June 2018, the patient is alive and physically active with a Karnofsky scale index of 90%. Imatinib administration is continued, no significant side effects are observed, and no surgical procedures are required.

## DISCUSSION

Tyrosine kinase inhibitors (TKIs) represent a class of target drugs that have been widely integrated into clinical practice since the beginning of the twenty-first century (Vergoulidou 2015). Protein tyrosine kinases (TKs) play key roles in the development and progression of cancer by acting as major components of various intracellular signaling pathways. These enzymes actively participate in many intracellular processes, including proliferation, metabolism, angiogenesis, differentiation, and apoptosis (Zhang and Liu 2002; Paul and Mukhopadhyay 2004). TKIs act by inhibiting TKs, thereby modulating downstream signaling.

Imatinib is a TKI, which targets pathological fusion enzyme BCR-ABL, platelet-derived growth factor receptors (PDGFRs), KIT, and several other TKs (Druker et al. 2001; Matei et al. 2004; Miselli et al. 2007; Ren et al. 2011). Imatinib is a derivative of 2-phenylaminopyrimidine and acts through blocking of the ATP-binding domain of TKs, thus preventing their phosphorylation and subsequent activation (Iqbal and Iqbal 2014). Imatinib monotherapy is FDA-approved for the treatment of Philadelphia chromosome–positive chronic myeloid leukemia and *Kit*-positive unresectable malignant gastrointestinal stromal tumors (Demetri et al. 2002; Peng et al. 2005; Berman et al. 2013). The Phase 2 clinical trial of imatinib monotherapy in epithelial ovarian cancer was terminated because of the absence of an objective response (NCT00510653). The combination of imatinib and paclitaxel in recurrent epithelial ovarian cancer was studied in trial NCT00840450. Twelve-month progression-free survival was obtained for only 17% of participants. Thus, there was no evidence of imatinib efficacy in ovarian cancer. However, several other TKIs showed promising efficacy in treatment of epithelial ovarian cancer (Ntanasis-Stathopoulos et al. 2016). Several studies investigated the efficacy of imatinib in ovarian GCT. The results obtained in cell lines were controversial (Chu et al. 2008; Jamieson and Fuller 2015); however, a previous case reported the benefit of imatinib in GCT of the ovary (Raspagliesi et al. 2011).

Here, we report a case of adult-type recurrent ovarian GCT, successfully treated with imatinib monotherapy. Although the disease progressed during best supportive care, imatinib treatment resulted in a prolonged stabilization of the disease.

Importantly, the prescription of imatinib was based on the bioinformatical analysis of gene expression data from the patient's tumor biopsy (Oncobox platform). Moreover, sorafenib treatment, which was also suggested by Oncobox, resulted in a partial tumor response and was terminated only because of significant side effects.

We conclude that a personalized approach for TKI prescription in ovarian cancer is needed. The selection of patients who may potentially benefit from imatinib or other TKI treatment may be based on the molecular profiling of tumor biopsies and further bioinformatical analysis. However, further extended clinical trials are required for validation and adjustment of clinical indications for this particular bioinformatical platform Oncobox.

## MATERIALS AND METHODS

An FFPE block with >80% of tumor cells was analyzed. We extracted RNA from five 250-µm-thick simultaneously made sections of this block. DNA was extracted from the FFPE tissue using the AnaPrep FFPE DNA extraction kit following the manufacturer's instruction. Whole-exome DNA was captured from total genomic DNA using the SeqCap EZ System from NimbleGen according to the manufacturer's instructions. Briefly, genomic DNA was sheared and size selected to roughly 200–250 base pairs and the ends were repaired and ligated to specific adapters and multiplexing indexes. Fragments were then incubated with SeqCap biotinylated DNA baits followed by the ligation-mediated PCR, and the RNA–DNA hybrids were purified using streptavidin-coated magnetic beads. The RNA baits were then digested to release the targeted DNA fragments, followed by a brief amplification of 15 or fewer PCR cycles. Sequencing was performed using Illumina HiSeq 3000. The reads were aligned with BWA-MEM. Sequencing coverage table is available in Supplemental Table S4. Mutation calling was performed using the Picard and Genome Analysis Toolkit.

The gene expression profile in the mixed sample was analyzed using the microarray platform CustomArray^Inc^ (USA). The Manufacturer's protocol was modified by adding to the amplification reaction dNTP mix containing biotinylated dUTP, resulting in a final proportion of dTTP/biotin-dUTP as 5/1. Hybridization was performed according to CustomArray ElectraSense Hybridization and Detection protocol. The hybridization mix contained 2.5 µg of labeled DNA library, 6× SSPE, 0.05% Tween 20, 20 mM EDTA, 5× Denhardt's solution, 100 ng/µL sonicated calf thymus gDNA, and 0.05% SDS. The hybridization mix was incubated with chip overnight at 50°C. Hybridization efficiency was detected electrochemically using the CustomArray ElectraSense Detection Kit and ElectraSense 4X2K/12K Reader.

Gene expressions of more than 3000 human genes were profiled and deposited at GES under accession ID GSE112579. The analysis of gene expression peculiarities was performed based on comparison with four samples of healthy ovarian tissues (samples of normal ovary from data set GSE6008). Gene expression profiles were pooled and quantile-normalized using the R statistical programming language and “preprocessCore” library.

The profiling of intracellular signaling pathways altered in the patient's tumor tissue when compared to normal was performed using the Oncobox bioinformatical platform. The Oncobox system is capable of modeling the drug's ability to block pathological changes in molecular pathways and simultaneously block gene products with a pathological increase in the expression level. In contrast to other known analogs, the Oncobox platform uses the parameter of the balanced efficiency score (BES) for each drug as a target drug efficiency measure. Wherein, the data on molecular pathway activity in a test sample and the data on expression levels of gene products—targets of a certain drug—are simultaneously used for the BES calculation. The BES value is calculated according to the formula

$${\mathrm{BES}}_{d}=a\times {\mathrm{DES}}_{d}^{\mathrm{MP}}+b\times {\mathrm{DES}}_{d}^{\mathrm{TG}},$$

in which *d* is the target drug under investigation; *a* and *b* are the weight coefficients varying from −1 to 1.5 depending on the target drug type; the drug efficiency index for molecular pathways DES^MP^_*d*_ is calculated based on the activity levels for molecular pathways containing molecular targets of drug *d*; and the drug efficiency score for target genes DES^TG^_*d*_ is calculated based on levels of expression of individual gene products.

To calculate DES^MP^, the formula

$${\mathrm{DES}}_{d}^{\mathrm{MP}}=\underset{t}{\sum }{\mathrm{DTI}}_{dt}\times \underset{p}{\sum }{\mathrm{PAL}}_{p}\times {\mathrm{AMCF}}_{p}\times {\mathrm{NII}}_{tp}$$

is used, in which *d* is the unique identifier of the target drug; *t* is the unique identifier of the gene product, the target of drug *d*; *p* is the unique identifier of the signaling pathway; PAL*_p_* is the molecular pathway *p* activation strength; and the discrete value AMCF (activation-to-mitosis conversion factor) is to be determined as follows:

$$\mathrm{AMCF}=\{\begin{array}{l} \text{1, when the activation of a pathway facilitates cell survival, growth, and division;}\\ \text{0, when there are no data as to whether the molecular pathway activation facilitates}\\ \text{cell survival, growth, and division, or when such data available to the researcher are conflicting;}\\ \;-1,\text{when the activation of a pathway prevents cell survival, growth, and division.} \end{array}$$

The discrete value DTI (drug–target index) is defined as

$${\mathrm{DTI}}_{dt\;}=\;\{\begin{array}{l} 0,\;\;\;\mathrm{when}\;\mathrm{drug}\;d\;\mathrm{does}\;\mathrm{not}\;\mathrm{affect}\;\mathrm{gene}\;\mathrm{product}\;t;\\ 1,\;\mathrm{when}\;\mathrm{drug}\;d\;\mathrm{affects}\;\mathrm{gene}\;\mathrm{product}\;t.\;\;\;\;\;\; \end{array}$$

The discrete value NII (node involvement index) is defined as

$${\mathrm{NII}}_{tp\;}=\;\{\begin{array}{l} 0,\;\mathrm{there}\;\mathrm{is}\;\mathrm{no}\;\mathrm{gene}\;\mathrm{product}\;t\;\mathrm{in}\;\mathrm{pathway}\;p;\\ 1,\;\mathrm{there}\;\mathrm{is}\;\mathrm{gene}\;\mathrm{product}\;t\;\mathrm{in}\;\mathrm{pathway}\;p.\;\; \end{array}$$

To calculate DES^TG^, use

$${\mathrm{DES}}_{d}^{\mathrm{TG}}=\underset{t}{\sum }{\mathrm{DTI}}_{dt}\times \underset{p}{\sum }\ln({\mathrm{CNR}}_{t})\times {\mathrm{ARR}}_{tp}\times {\mathrm{AMCF}}_{p}\times {\mathrm{NII}}_{tp},$$

in which *d* is the unique identifier of the target drug; *t* is the unique identifier of the gene product, molecular target of drug *d*; *p* is the unique identifier of the signaling pathway; CNR*_t_* (case-to-normal ratio) is the ratio of the expression levels of the protein-coding gene *t* in the test sample to the norm (averaged expression level for a control group); ln is the natural logarithm; the definitions of DTI*_d_*_,*t*_, AMCF_p_, and NII are similar to those given above. The discrete value ARR*_tp_* (activator/repressor role) is defined for a gene product *t* in the pathway *p* as follows and deposited into the molecular pathway database:

$${\mathrm{ARR}}_{tp}=\;\{\begin{array}{l} -1,\;\mathrm{gene}\;\mathrm{product}\;t\;\mathrm{is}\;\mathrm{repressor}\;\mathrm{of}\;\mathrm{pathway}\;p;\\ -0.5,\text{gene product}\;t\;\text{is rather repressor than activator of pathway}\;p;\\ 0,\;\mathrm{activator}/\mathrm{repressor}\;\mathrm{role}\;\mathrm{of}\;\mathrm{gene}\;\mathrm{product}\;t\;\mathrm{in}\;\mathrm{pathway}\;p\;\mathrm{is}\;\mathrm{unclear}\;\mathrm{or}\\ \mathrm{unknown};\\ 0.5,\mathrm{gene}\;\mathrm{product}\;t\;\mathrm{is}\;\mathrm{rather}\;\mathrm{activator}\;\mathrm{than}\;\mathrm{repressor}\;\mathrm{of}\;\mathrm{pathway}\;p;\\ \;1,\;\mathrm{gene}\;\mathrm{product}\;t\;\mathrm{is}\;\mathrm{activator}\;\mathrm{of}\;\mathrm{pathway}\;p. \end{array}$$

To calculate the BES for drug *d*, weight coefficients *a* and *b* are used, which differ depending on the drug type.

For low-molecular TKIs (nibs), both weight coefficients are equal to 0.5, representing the equal significance of the target molecular pathway activation and target gene expression levels in the pathological tissue sample tested. This is related to the nibs’ capability of blocking their molecular targets and thus inhibiting their activities, as well as modulating the cell signaling via related molecular pathways. For hormones, both weight coefficients are equal to −0.5, because they activate but do not inhibit their molecular targets and act accordingly also on their target molecular pathways. For antihormones, coefficients are equal to 0.5 again, which is because of their inhibition effect on their molecular targets, hormone products, and on the respective molecular pathways. For retinoids, both coefficients are equal to 0.5 because these drugs bind retinoic acid receptors and activate a number of dependent molecular pathways. For rapalogs (rapamycin analogs), both coefficients are equal to 0.5 because they demonstrate their inhibition effect by directly binding with their molecular targets and act accordingly on the relevant molecular pathways. For mibs (proteasome inhibitors), both coefficients are equal to 0.5 because these drugs demonstrate the inhibition effect when binding with their molecular targets and act accordingly on the relevant molecular pathways and proteasome signaling. For VEGF blocking agents, *a* = 0 and *b* = 1, because these drugs directly block the VEGF molecules in the blood flow while not binding with the molecular targets inside the cell or on the cell surface and, therefore, do not directly affect the intracellular signaling. For monoclonal antibodies that bind with their molecular targets on the cell surface (mAbs), *a* = 0 and *b* = 1, as their main mode of action consists in activation of immune cytotoxic response against the cells having bound mAb molecules on their surface and does not deal with strong modulation of signaling by affecting molecular pathways. Killer mAbs consist of antibodies against molecular targets on the cell surface chemically bound with cytotoxic agents. When binding with their targets on the cell surface, the killer mAbs kill these cells, thus demonstrating therapeutic mechanism not related to intracellular molecular pathway activation. For them, *a* = 0 and *b* = 1.5; in this case, the increased coefficient *b* represents proprietary high cytotoxic activities of these drugs. For drugs blocking de novo tubulin polymerization, *a* = 0 and *b* = 1; this represents the indefinite function of many targeted pathways for these drugs in cell survival and proliferation, as well as their direct inhibitory effect on their molecular targets. The same coefficients are also set for histone deacetylase inhibitors for the same reasons concerning their mechanism of action. For DNA-alkylating agents, *a* = 0 and *b* = −1, reflecting the indefinite functions of the majority of targeted pathways for cell survival and proliferation, as well as the direct inhibitory effect of these drugs on DNA repair proteins that target the alkylated DNA (reflected by the coefficient *b* = −1). For immunotherapeutic drugs, both coefficients are equal to 0.5, because of the dependence of their effect on the availability of both direct molecular targets and molecular pathway activation profiles related to tumor infiltration with lymphocytes. Similarly, the poly-ADP ribose polymerase blocking drugs inhibit DNA repair and depend on both availability of direct molecular targets and on the activities of targeted molecular pathways. This is reflected by both coefficients *a* and *b* being equal to 0.5.

## ADDITIONAL INFORMATION

### Data Deposition and Access

Gene expression data derived from the patient's tumor tissue were deposited at the GEO (https://www.ncbi.nlm.nih.gov/geo/) under accession number GSE112579. Whole-exome sequencing data were deposited to NCBI SRA under accession number PRJNA503667.

### Ethics Statement

The patient provided informed written consent for gene expression analysis and whole-exome sequencing of her sample and publication of this article. Gene expression profiling was approved by the Institutional Review Board (IRB) at Clinical Center Vitamed, Moscow, Russia, according to the principles of the Declaration of Helsinki.

### Author Contributions

E.V.P., M.P.B., D.O.A., and V.B. collected and interpreted patient data. E.V.P. and M.P.B. were involved in clinical management. M.V.S. and D.E.K. performed molecular analyses. L.M. analyzed and interpreted the MRI data. M.V.S., A.A.A., I.N.K., and E.V.P. wrote this manuscript.

### Competing Interest Statement

The authors have declared no competing interest.

## Supplementary Material

Supplemental Material
