# Supplementary material for: Personalized prescription of imatinib in recurrent granulosa cell tumor of the ovary: case report
Source: Cold Spring Harb Mol Case Stud. 2019 Apr;5(2):a003434. doi: 10.1101/mcs.a003434 (PMC6549576; doi:10.1101/mcs.a003434)
Supplement: Supplemental Material [file supp_5_2_a003434__index.html]

Personalized prescription of imatinib in recurrent granulosa cell tumor of the ovary: case report — Supplemental Material 

# Personalized prescription of imatinib in recurrent granulosa cell tumor of the ovary: case report

## Supplemental Material

- Supplemental\_Figure\_S1.jpg
- Supplemental\_Figure\_S2.jpg
- Supplemental\_Figure\_S3.jpg
- Supplemental\_Figure\_S4.jpg
- Supplemental\_Figure\_S5.jpg
- Supplemental\_Figure\_S6.jpg
- Supplemental\_Figure\_S7.jpg
- Supplemental\_Figure\_S8.jpg
- Supplemental\_Figure\_S9.jpg
- Supplemental\_Figure\_S10.jpg
- Supplemental\_Material.docx
- Supplemental\_Table\_S3.xlsx
- Supplemental\_Table\_S4.xlsx
- Supplemetnal\_Table\_S2\_PAL-DS.xlsx
