# Supplementary figures and images for: Personalized prescription of imatinib in recurrent granulosa cell tumor of the ovary: case report
Source: Cold Spring Harb Mol Case Stud. 2019 Apr;5(2):a003434. doi: 10.1101/mcs.a003434 (PMC6549576; doi:10.1101/mcs.a003434)

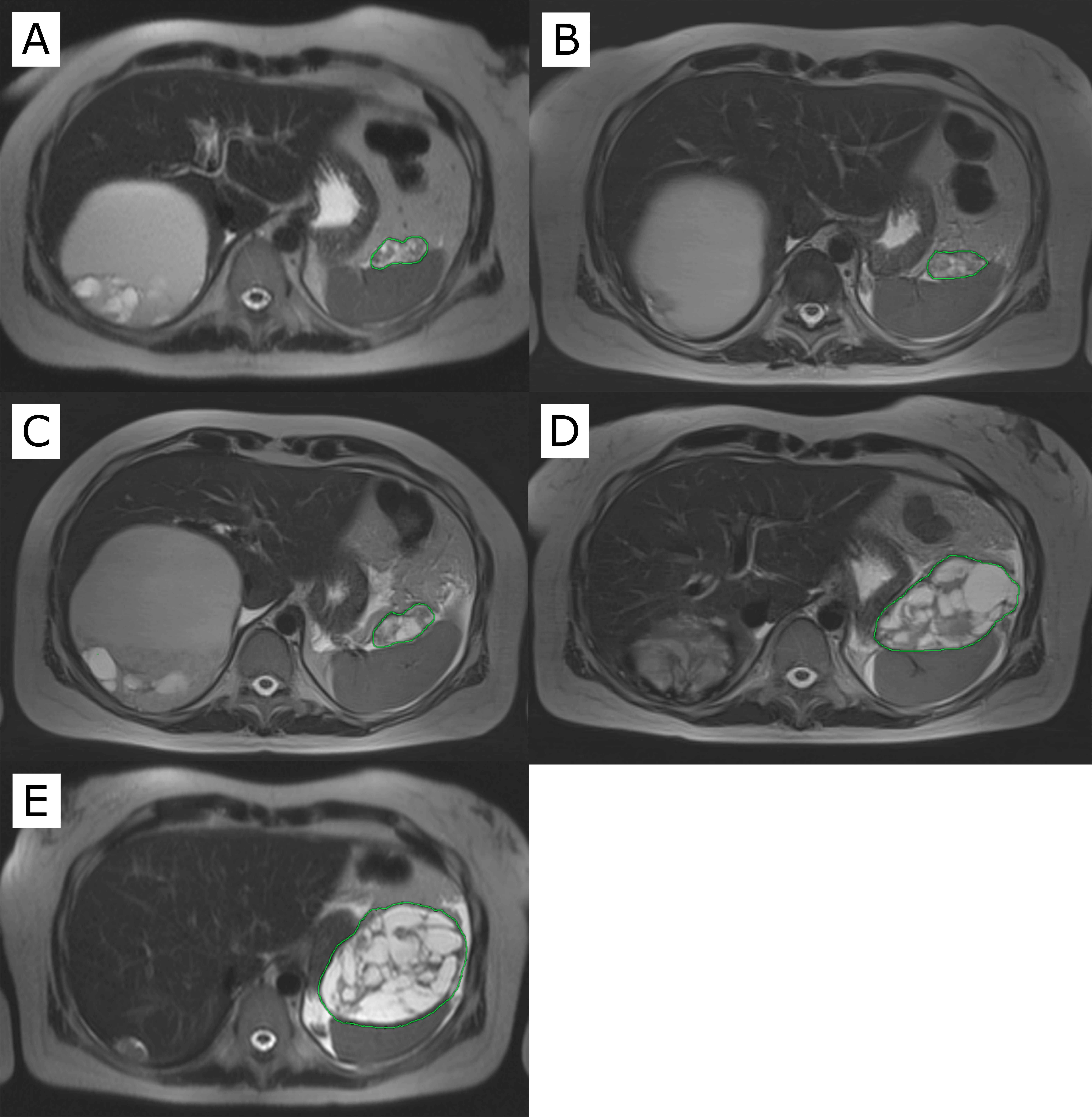

Supplement: Supplemental Material [file supp_mcs.a003434_Supplemental_Figure_S1.jpg]

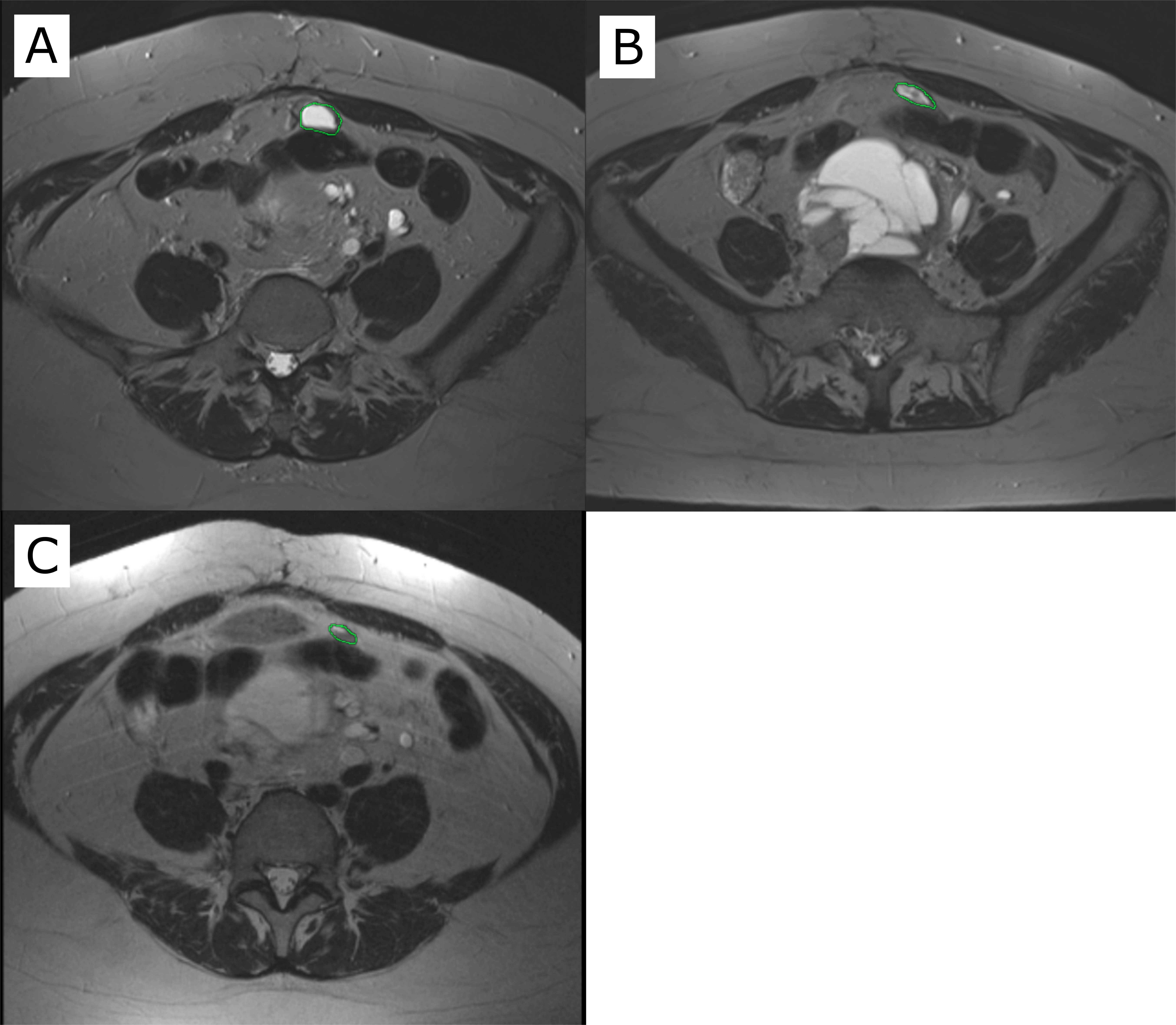

Supplement: Supplemental Material [file supp_mcs.a003434_Supplemental_Figure_S2.jpg]

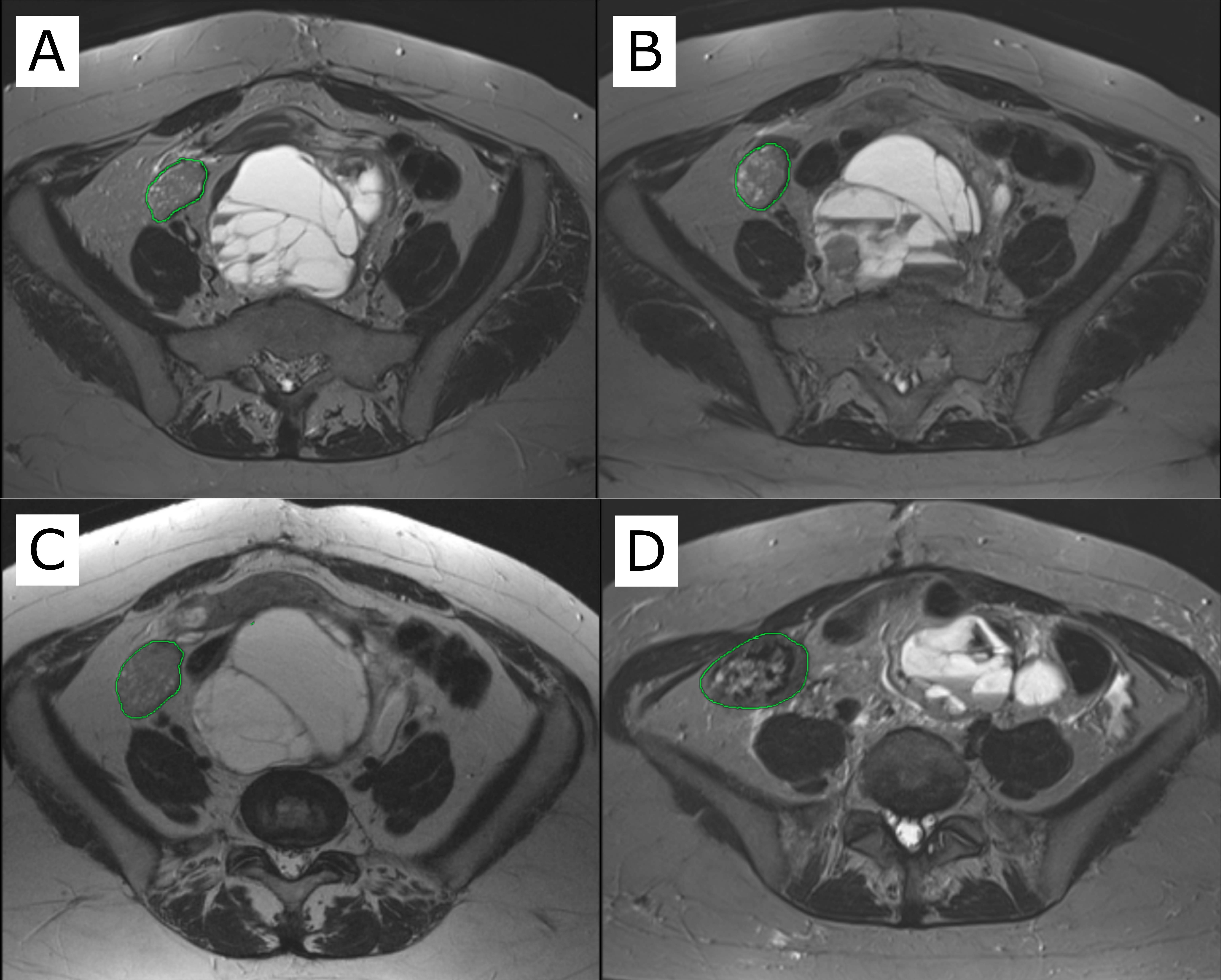

Supplement: Supplemental Material [file supp_mcs.a003434_Supplemental_Figure_S4.jpg]

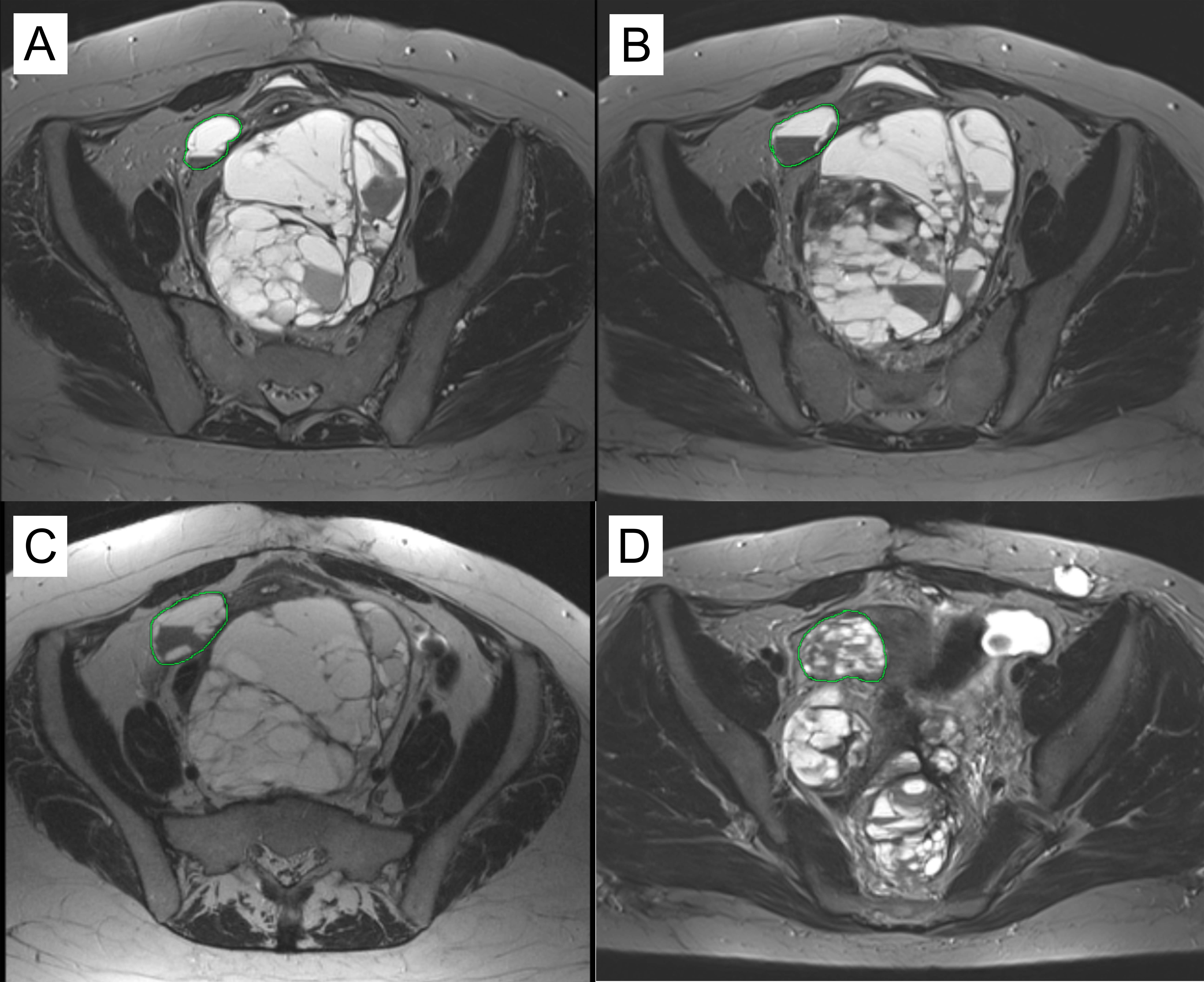

Supplement: Supplemental Material [file supp_mcs.a003434_Supplemental_Figure_S5.jpg]

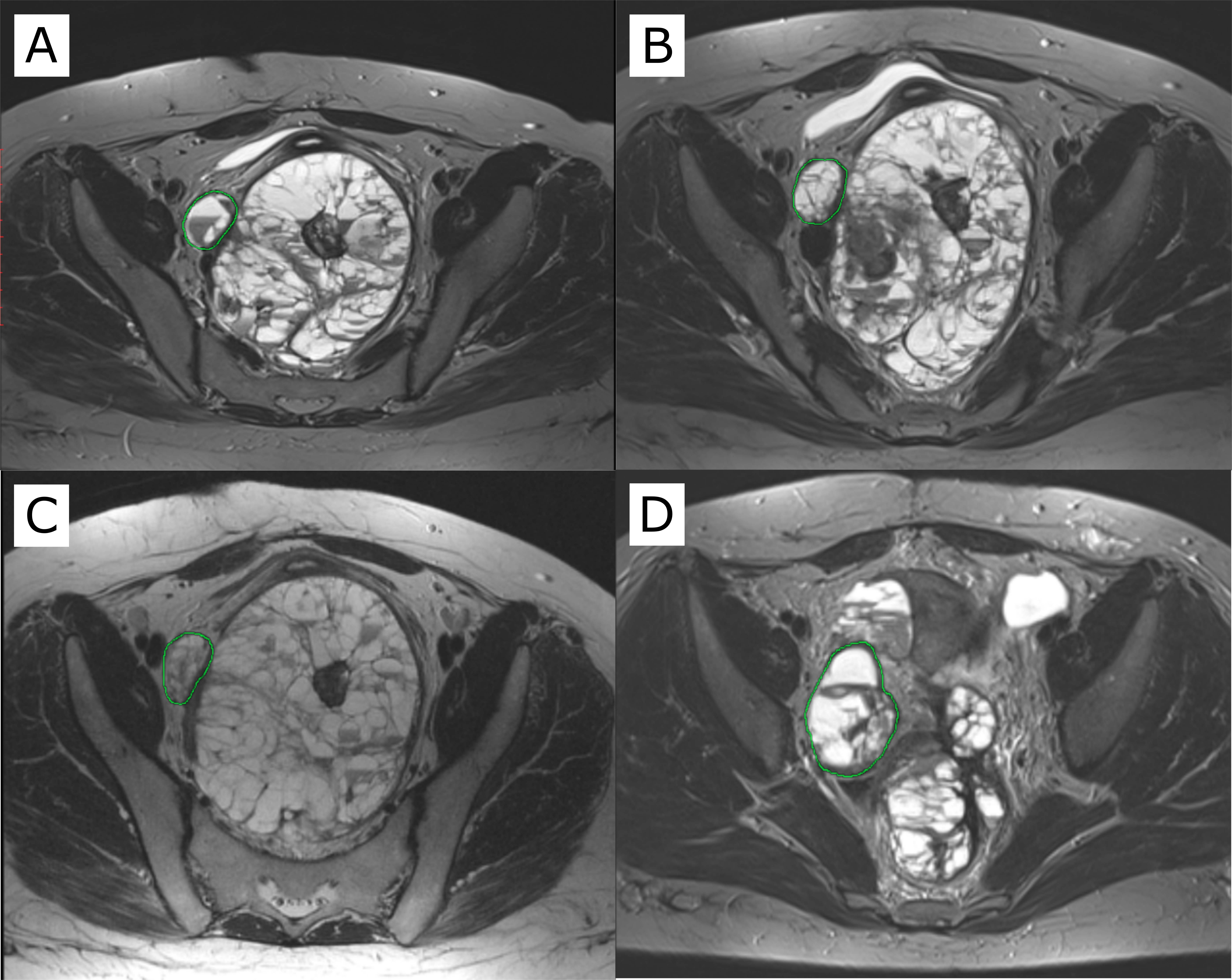

Supplement: Supplemental Material [file supp_mcs.a003434_Supplemental_Figure_S6.jpg]

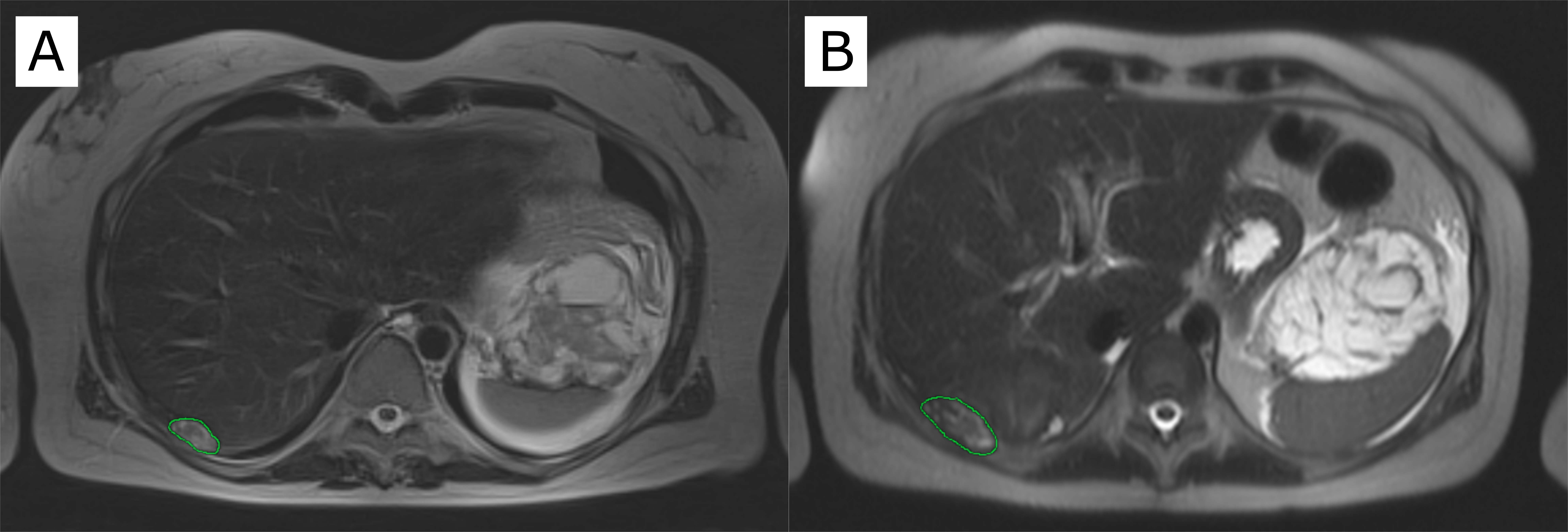

Supplement: Supplemental Material [file supp_mcs.a003434_Supplemental_Figure_S8.jpg]

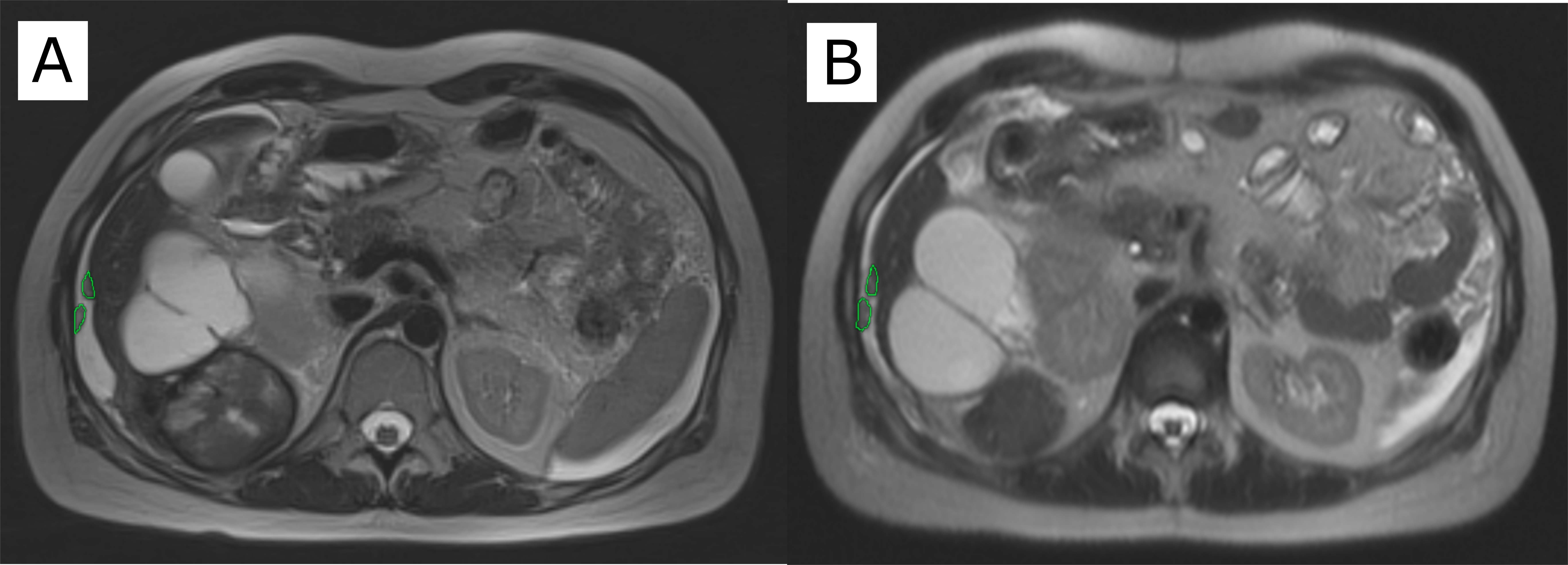

Supplement: Supplemental Material [file supp_mcs.a003434_Supplemental_Figure_S9.jpg]

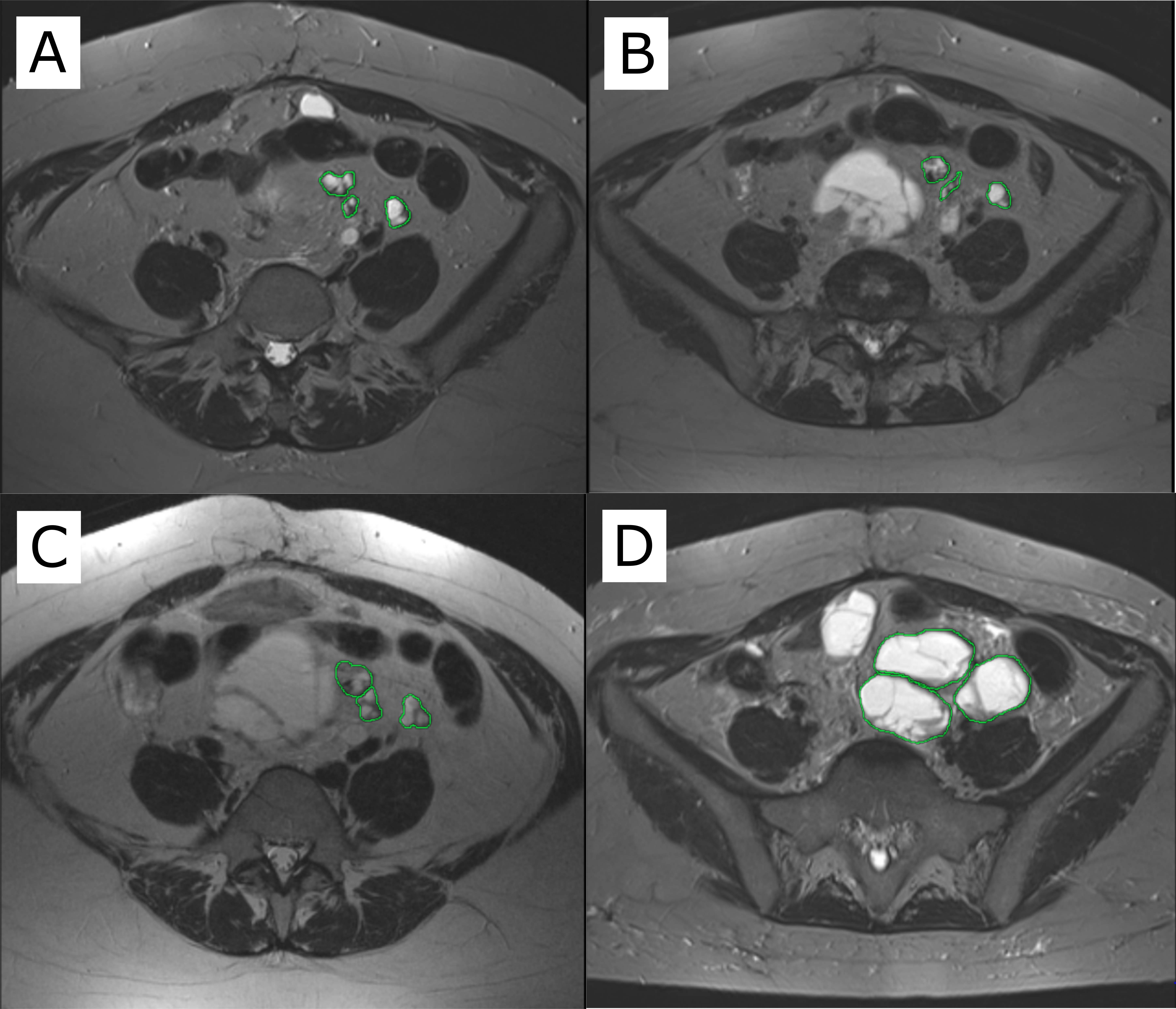

Supplement: Supplemental Material [file supp_mcs.a003434_Supplemental_Figure_S10.jpg]
