# Supplementary material for: Personalized prescription of imatinib in recurrent granulosa cell tumor of the ovary: case report
Source: Cold Spring Harb Mol Case Stud. 2019 Apr;5(2):a003434. doi: 10.1101/mcs.a003434 (PMC6549576; doi:10.1101/mcs.a003434)
Supplement: Supplemental Material [file supp_mcs.a003434_Supplemental_Material.docx]

**Supplementary Figure Legends.**

Figure S1. Neoplasm # 4. А – MRI from 03.2016, B - MRI from 06.2016, C – MRI from 09.2016, D – MRI from 10.2017, E – MRI from 03.2018.

Figure S2. Neoplasm # 5. А – MRI from 03.2016, B - MRI from 06.2016, C – MRI from 09.2016.

Figure S3. Neoplasm # 6. А – MRI from 03.2016, B - MRI from 06.2016, C – MRI from 09.2016, D – MRI from 10.2017, E – MRI from 03.2018.

Figure S4. Neoplasm # 7. А – MRI from 03.2016, B - MRI from 06.2016, C – MRI from 09.2016, D – MRI from 03.2018.

Figure S5. Neoplasm # 8. А – MRI from 03.2016, B - MRI from 06.2016, C – MRI from 09.2016, D – MRI from 03.2018.

Figure S6. Neoplasm # 9. А – MRI from 03.2016, B - MRI from 06.2016, C – MRI from 09.2016, D – MRI from 03.2018.

Figure S7. Neoplasm # 10. А – MRI from 03.2016, B - MRI from 06.2016, C – MRI from 09.2016, D – MRI from 10.2017, E – MRI from 03.2018.

Figure S8. Neoplasm # 11. А – MRI from 10. 2017, B – MRI from 03.2018.

Figure S9. Neoplasm # 12. А – MRI from 10. 2017, B – MRI from 03.2018.

Figure S10. Neoplasm # 13. А – MRI from 06.2016, B - MRI from 09.2016, C – MRI from 03.2018.

**Table S1. Neoplasms revealed in MRI, CT and USI examinations**

| **# Neoplasm** | **CT scan** | **USI** | **USI** | **USI** | **MRI** | **MRI** | **MRI** | **USI** | **USI** | **MRI** | **MRI** |
| --- | --- | --- | --- | --- | --- | --- | --- | --- | --- | --- | --- |
|  | **08.2013** | **09.2014** | **06.2015** | **01.2016** | **03.2016** | **06.2016** | **09.2016** | **12.2016** | **02.2017** | **10.2017** | **03.2018** |
| **#1** cystic-solid neoplasm on the lower contour of the liver (Fig. 3) | 3.8 × 2.5 cm | 10.5 × 8.2 cm | 10.5 × 8.2 cm | 9.0 × 7.0 cm | 10.4 × 10.2 × 12.6 cm | 12.6 × 11.5 × 14. cm | 12.3 × 11.2 × 15.2 cm | 11.0 × 12.0 × 14.0 cm | 15.5 × 12.0 cm | 6.7 × 8.5 × 8.3 cm | 6.1 × 7.8 × 8.1 cm |
| **#****2** сystic-solid multinodular formation in Douglas space, part of nodes with high-density content (Fig. 4) | 4.8 × 4.5 cm |  | 10.8 × 9.0 cm | 12.0 × 9.6 × 10 cm | 12.7 × 8.7 cm | 13.2 × 8.8 cm | 13.2 × 9.7 cm | - | - | Not investigated | presented by separate nodal formations up to 5.3 × 6.5 cm and 3.1 × 3.3 cm |
| **#3** cystic-solid neoplasm with tuberous contours in the right lateral channel (Fig. 5) | 6.8 × 5.8 cm | - | 7.0 × 6.5 cm | 5.6 × 5.4 cm | 3.3 × 5.3 × 4.3 cm | 4.6 × 6.4 × 4.8 cm | 4.5 × 5 × 4.3 cm | 8.4 × 6.6 cm | 9.0 × 7.5 cm | 7 × 8.2 × 9.4 cm | 8.2 × 9.2 × 9.9 cm |
| **#4** neoplasm at the splenic hilum (Fig. S1) | 3.4 × 2.4 cm | 6.7 × 4.8 cm | 4.2 × 3.5 cm | 2.7 × 2.3 cm | 1.6 × 3.5 cm | 1.8 × 3.8 cm | 2.1 × 4.3 cm | - | 5.0 × 3.3 cm | 5.2 × 9.3 cm | 7.1 × 10.3 cm |
| **#5** cystic node under the anterior abdominal wall, at the level of the entrance to the pelvis (Fig. S2) | - | - | - | - | 1.3 × 1.9 cm | 0.7 × 1.2 cm | 0.6 × 1.1 cm | - | - | Not investigated | was not determined reliably |
| **#6** neoplasm on the anterior contour of the gallbladder (Fig. S3) | - | - | - | - | 1.6 × 2.2 cm | 1.9 × 3.1 cm | 2.4 × 2.7 cm | - | - | 2.1 × 1.6 cm | 4.5 × 2.2 cm |
| **#7** neoplasm in the field of the right ovary (Fig. S4) | - | - | - | - | 3.4 × 2 cm | 3.1 × 2.1 cm | 3.9 × 2.7 cm | - | - | Not investigated | 4.8 × 2.7 cm |
| **#8** neoplasm in the field of the right ovary (Fig. S5) | - | - | - | - | 2.9 × 1.7 cm | 3.5 × 2.0 cm | 4.2 × 2.2 cm | - | - | Not investigated | 3.9 × 3 cm |
| **#9** neoplasms in Douglas pouch in the right contour (Fig. S6) | - | - | - | - | 3.5 × 2.1 cm | 3 × 1.9 cm | 3.1 × 2 cm | - | - | Not investigated | 5.4 × 3.9 cm |
| **#10** multichamber cystic node in the sub-hepatic space (Fig. S7) | - | - | - | - | 4.0 × 2.0 cm | 4.3 × 2.6 cm | 4.3 × 2.9 cm | - | - | 7.6 × 4.3 cm | 9.3 × 6.9 cm |
| **#11** Sub-diaphragmally on the right, on the posterior contour of the right lobe of the liver, a cystic-solid node  (Fig. S8) | - | - | - | - | - | - | - | - | - | 1.2 × 2.4 cm | 1.6 × 2.5 cm |
| **#12** hypodense nodes along the peritoneum in the perihepatic spatium (Fig. S9) | - | - | - | - | - | -- | - | - | - | 8 mm | up to 10 mm |
| **#13** cystic formations to the left by the way of the common iliac vessels (Fig. S10) | - | - | - | - | up to 15 mm | up to 12 mm | 2.5 cm | - | - | Not investigated | 4.9 × 2.5 cm |
| **Sum of the diameters** | 34 | 30.2 | 59.7 | 54 | 99.7 | 112.1 | 113.9 (total)  73.7 (for comparison lesions investigated at 10.2017) | 52 | 52.3 | 82.6 | 94.7 |
